# Cutaneous Metastatic Lesions as a Rare Manifestation of Colorectal Adenocarcinoma: A Case Report

**DOI:** 10.7759/cureus.102895

**Published:** 2026-02-03

**Authors:** Jeevan Rivera-Díaz, Sofía Laguna Rocafort, Jose Rabelo-Cartagena

**Affiliations:** 1 School of Medicine, Universidad Central del Caribe, Bayamón, PRI; 2 Dermatology, VA Caribbean Healthcare System, San Juan, PRI

**Keywords:** cdx2, ck20, colorectal adenocarcinoma, colorectal cancer, cutaneous metastases, satb2, skin lesion, skin metastasis

## Abstract

Cutaneous metastasis is an uncommon manifestation of colorectal cancer, usually occurring in an advanced disease state. We present a case of a 93-year-old man with a history of multiple non-melanoma skin cancers who was previously diagnosed with primary adenocarcinoma of the colon. The patient presented with three nodules on his upper back that developed over a few weeks. Physical examination revealed two erythematous, firm, dome-shaped nodules without discharge, and a third, smaller, flesh-colored, and non-ulcerated lesion. No palpable regional lymphadenopathy was noted. Two punch biopsies were performed on representative lesions. Histopathologic analysis demonstrated irregular glandular structures infiltrating the dermis, composed of atypical mucin-producing columnar cells with abundant eosinophilic cytoplasm and extracellular mucin deposits interspersed among collagen fibers. Immunohistochemical staining was positive for CDX2, CK20, and SATB2, and negative for CK7, findings consistent with metastatic colorectal adenocarcinoma. The patient’s clinical course was consistent with advanced disease, and he passed away approximately three months after the onset of cutaneous lesions. This case highlights the importance of recognizing cutaneous metastases as a potential indicator of systemic spread in patients with a history of colorectal cancer. Prompt dermatologic evaluation, histopathologic confirmation, and multidisciplinary coordination are crucial for accurate staging and effective care. Greater awareness and standardized guidelines are necessary to enhance the diagnostic approach and management of this rare yet clinically significant manifestation.

## Introduction

Colorectal cancer (CRC) is the third most diagnosed malignancy and the second leading cause of cancer-related death in the United States [1], with an annual incidence of 37.1 new cases and 12.9 deaths per 100,000 individuals [2]. Globally, it is the second most fatal cancer [3]. Metastatic spread may occur via lymphatic dissemination, hematogenous routes, peritoneal seeding, or intraluminal extension [4]. The most frequent metastatic sites include the liver, lungs, and central nervous system [5].

Cutaneous metastases (CM) represent an uncommon manifestation of internal malignancies, occurring in fewer than 5% of patients with CRC [6]. The skin accounts for approximately 0.7-9.0% of all malignant metastases, making it an uncommon site of secondary involvement [6]. In 2025, 100 cases of CM from CRC were reported in the literature [6]. Although rare, their recognition is clinically significant, as they often signal advanced disease and portend a poor prognosis [7]. Skin metastases from colorectal origin typically occur near the primary tumor site, though distant cutaneous involvement has also been reported [5,8]. Usually, they affect individuals aged 50-70 years and are more common in men [7].

Clinically, these lesions can resemble a variety of other dermatologic entities [9]. CM may present as solitary or multiple nodules, erythematous plaques, ulcerations, or inflammatory and telangiectatic lesions, making diagnosis challenging [4,10]. Due to their non-specific presentation and low prevalence, CM are frequently misdiagnosed or underreported [11]. We present a case of a 93-year-old man with a history of colorectal adenocarcinoma who developed three cutaneous metastatic lesions on the back.

## Case presentation

A 93-year-old man with a past medical history notable for basal cell carcinoma, squamous cell carcinoma, and primary adenocarcinoma of the colon presented for dermatologic evaluation of newly developed reddish nodules on his back. Two years prior, he was diagnosed with a microsatellite-stable, well-differentiated adenocarcinoma (pathologic stage pT3N1a) of the ascending colon, positive for BRAF mutation, and negative for KRAS and NRAS mutations for which he underwent a right hemicolectomy with ileotransverse anastomosis due to a 95% occlusion [12]. At that time, the patient refused adjuvant therapy and opted for at-home hospice care. The newly developed skin lesions appeared a few weeks prior to evaluation and had been treated with antibiotics for the past 20 days, initially diagnosed as cellulitis, with no improvement. The patient denied pruritus, bleeding, fever, or other constitutional symptoms.

**Figure 1:**
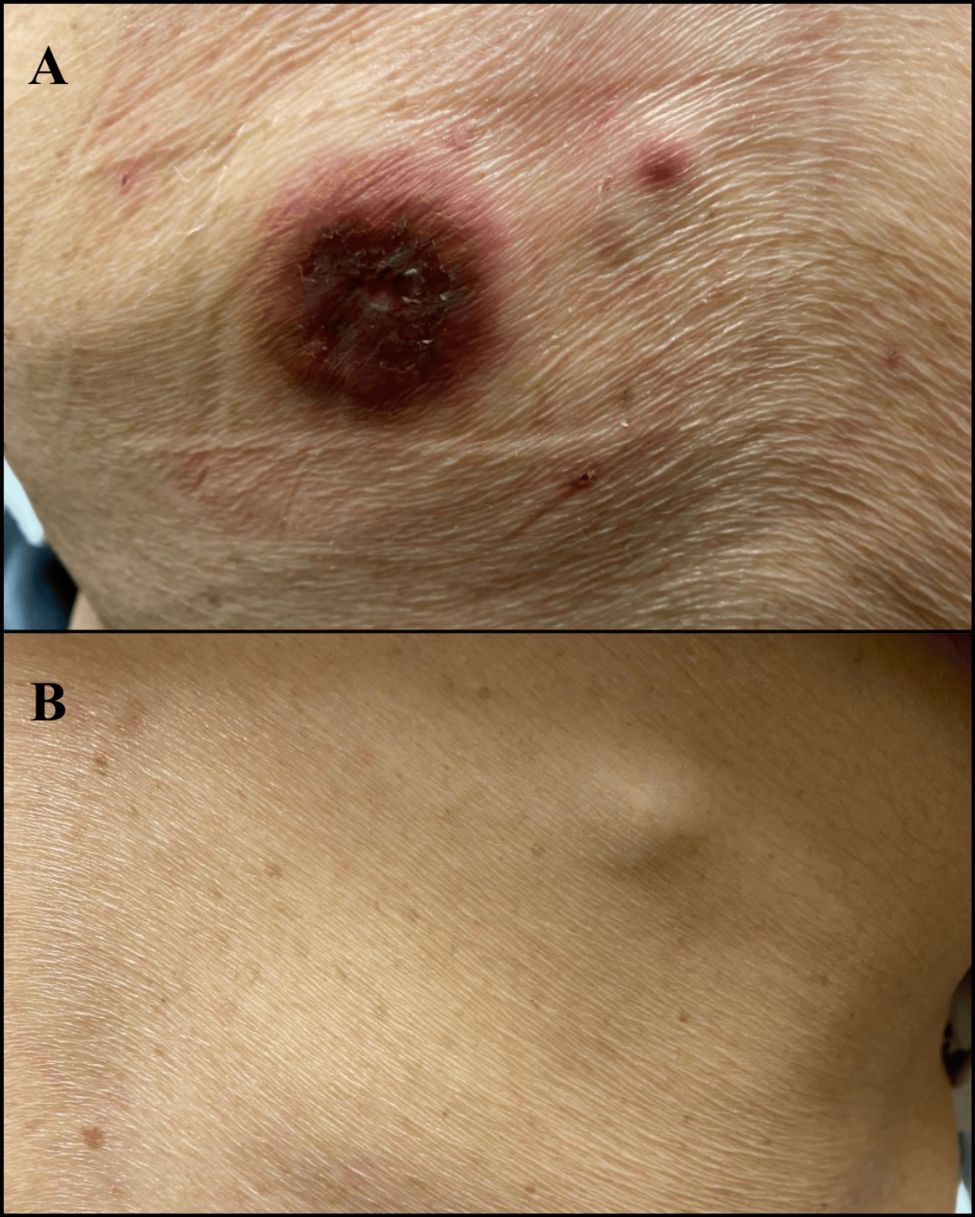
Clinical presentation of cutaneous metastases from colorectal adenocarcinoma. (A) Firm, dome-shaped, violaceous lesions in the left lateral and medial back. (B) Smaller, flesh-colored, and non-ulcerated nodule in the right lateral back.

Physical examination revealed two erythematous, firm, dome-shaped nodules on the left lateral and medial upper back, with tenderness but no discharge (Figure 1A). Moreover, a third, smaller, flesh-colored, and non-ulcerated lesion was also present on the right lateral upper back (Figure 1B). No palpable regional lymphadenopathy was noted. Punch biopsies were obtained from the erythematous lesions in the lateral (4.1x4.3 cm) and medial (0.6x0.4 cm) back.

**Figure 2:**
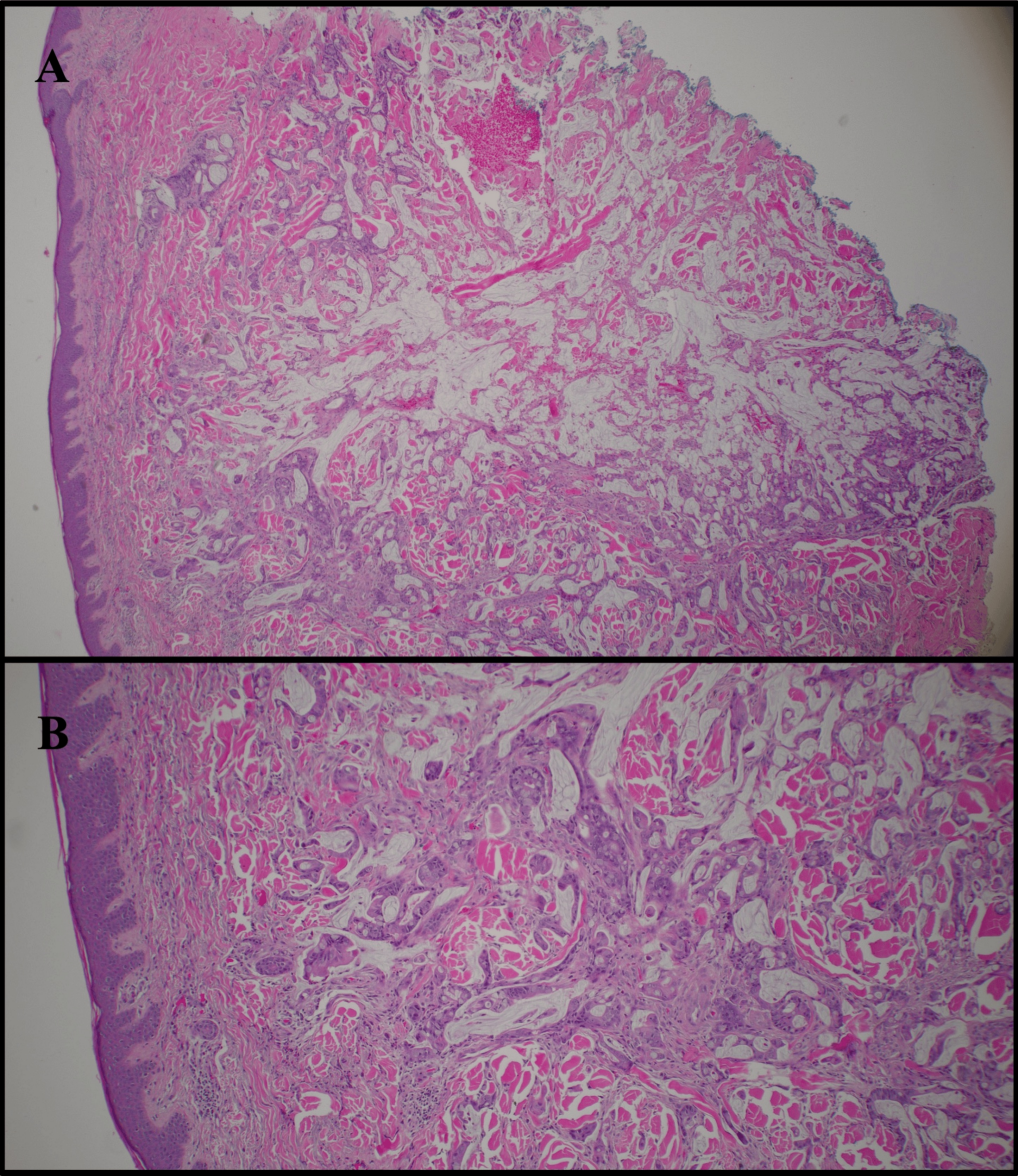
Histopathologic features of cutaneous metastasis from colorectal adenocarcinoma. (A) 10x view showing irregular malignant glandular structures within the dermis. (B) 20x view showing infiltrative tumor growth within a fibrotic dermal stroma.

**Figure 3:**
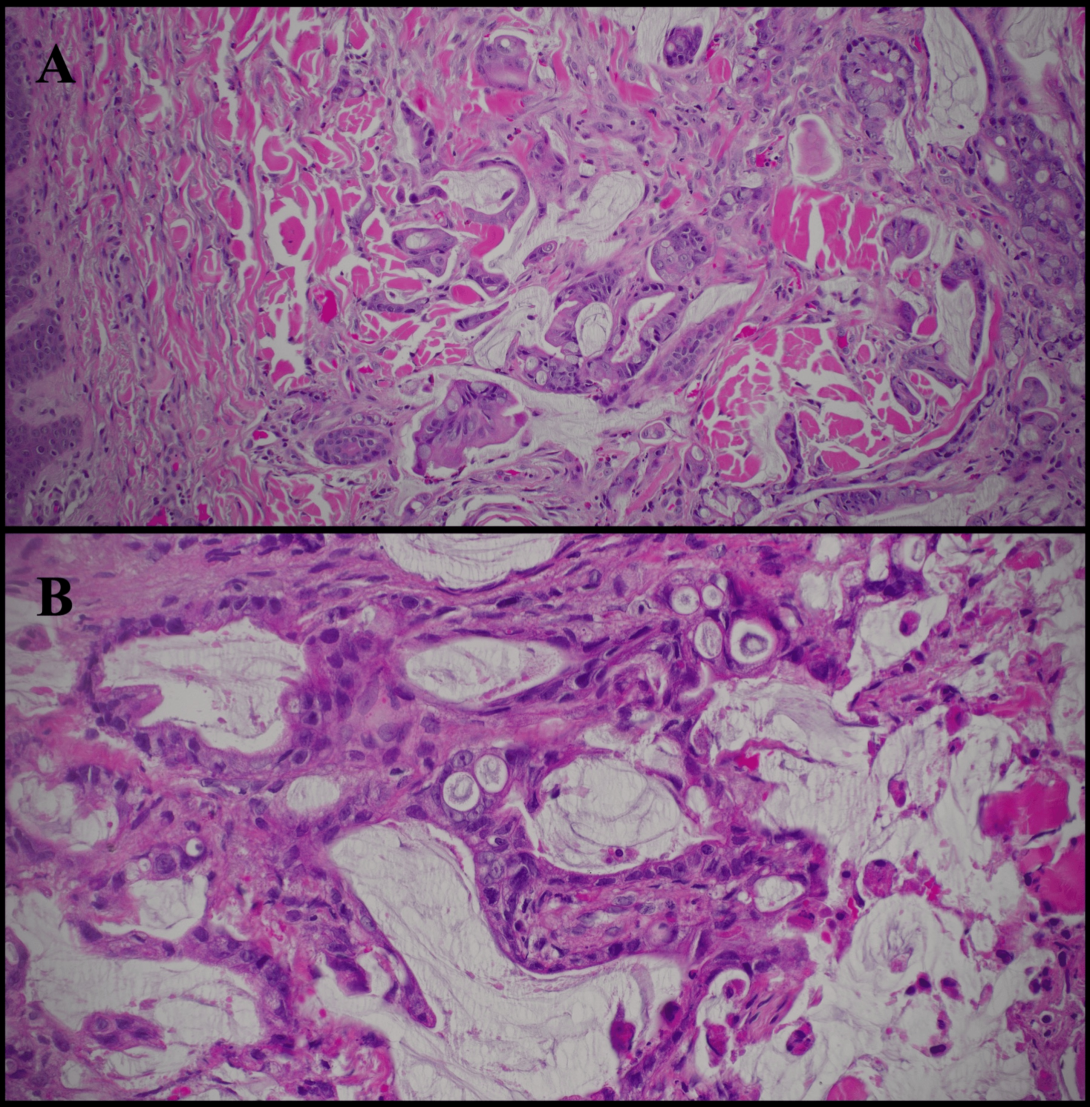
Overview of dermal involvement by metastatic colorectal adenocarcinoma. (A) 40x view demonstrating diffuse dermal infiltration by malignant glands. (B) 100x view highlighting infiltrative glandular architecture within the dermis.

**Figure 4:**
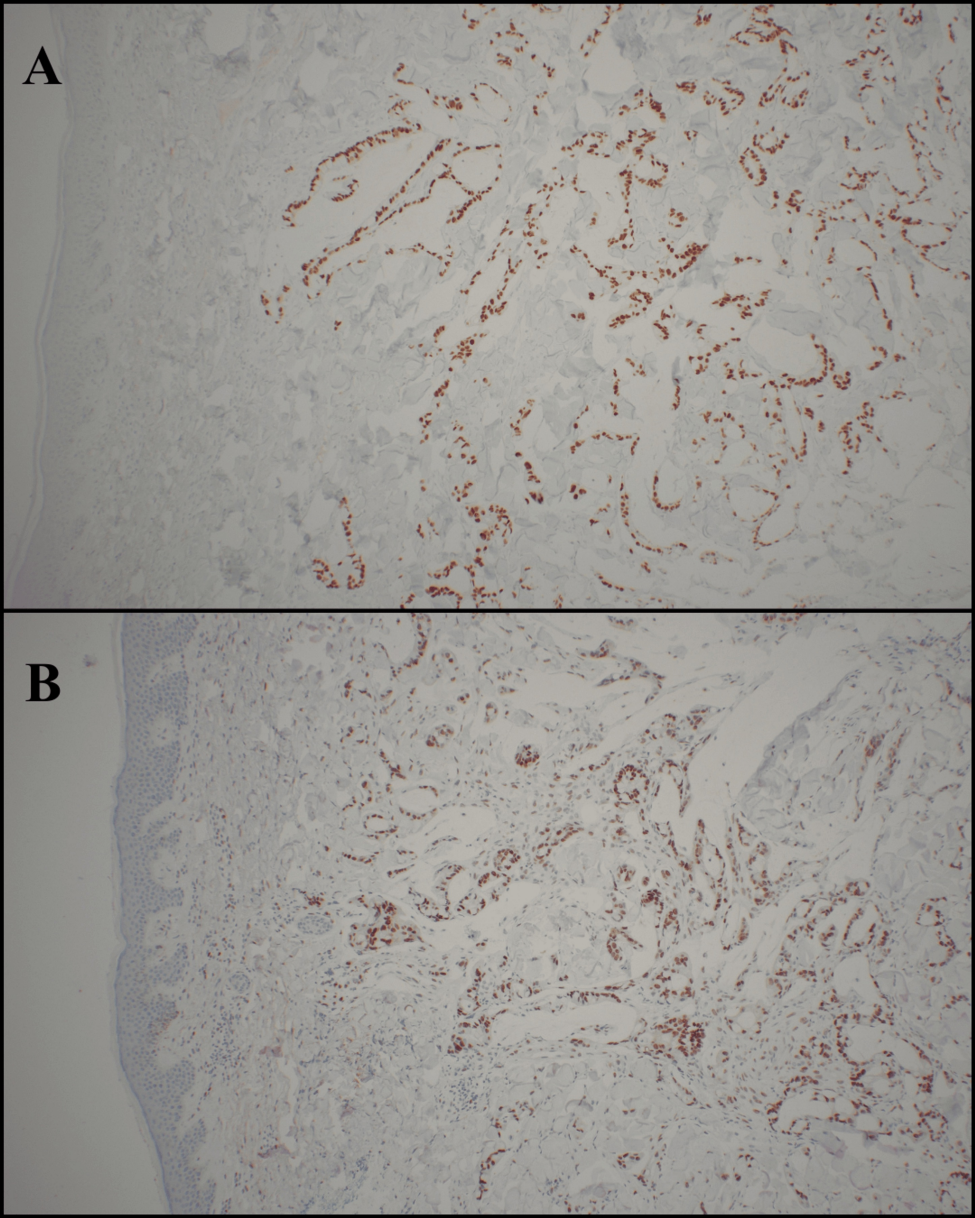
Immunohistochemical profile confirming colorectal origin of the cutaneous metastasis. (A) 20x view positive for CDX2 immunostaining. (B) 20x view positive for SATB2 immunostaining.

Histopathologic examination demonstrated a malignant epithelial neoplasm composed of irregular glandular structures infiltrating the dermis. The glands were lined by atypical mucin-producing columnar cells with abundant eosinophilic cytoplasm. Pools of extracellular mucin were noted interspersed among collagen fibers (Figures 2A, 2B, 3A, 3B). Immunohistochemical staining revealed tumor cells positive for CDX2, CK20, and SATB2, and negative for CK7, consistent with metastatic colorectal adenocarcinoma (Figures 4A, 4B). Upon diagnosis of cutaneous metastases (CM), family members opted to refuse further evaluation and therapeutic intervention and prioritize the patient’s quality of life.

## Discussion

Typically, CM appears late in the disease course but can occasionally serve as the first indication of an underlying internal malignancy [7]. Their presence indicates disseminated disease and is associated with poor prognosis, with reported median survival times ranging from three to six months following diagnosis [7,13]. This aligns with our patient’s clinical course, as he expired approximately three months after the onset of his cutaneous lesions. Clinically, CM often presents as painless, firm, flesh-colored or erythematous nodules, either solitary or multiple [14]. In this case, the patient presented with two firm, erythematous nodules and one non-ulcerated lesion on the upper back. CM often presents in a non-specific manner, leading to misdiagnosis [11]. This aligns with the present case, as the lesions were initially treated as cellulitis with antibiotic therapy.

Systemic diseases and other conditions with benign or malignant skin involvement should be included in the differential diagnosis of skin lesions that suggest CM [7]. Given the non-specific clinical appearance of CM, histopathologic and immunohistochemical evaluation are essential for diagnosis. Colorectal adenocarcinoma metastases typically demonstrate positivity for cytokeratin 20 (CK20) and caudal-type homeobox 2 (CDX2) and negativity for cytokeratin 7 (CK7), distinguishing them from other epithelial malignancies [15,16]. SATB2, a nuclear transcription factor involved in osteoblastic and colorectal differentiation, is the most specific marker for colorectal origin, with reported sensitivity ranging from 80% to 97% [17]. In our patient, the characteristic immunoprofile (CDX2+, CK20+, SATB2+, CK7-) confirmed the diagnosis of metastatic colorectal adenocarcinoma.

Advances in colorectal cancer (CRC) screening and treatment have markedly enhanced stage-specific survival outcomes. The five-year relative survival rate is approximately 90% for early-stage disease (stage I), whereas it decreases to around 14% for advanced-stage CRC (stage IV) [18]. Yet, current American and European guidelines do not specifically address CM management. Moreover, international recommendations lack a standardized, unified approach to treating these lesions, regardless of the underlying primary cancer type [6].

## Conclusions

It is crucial to maintain a high index of suspicion for CM in patients with a history of CRC who present with new or atypical skin findings. This case highlights the crucial role of histopathologic and immunohistochemical evaluation, particularly the use of markers such as CK20, CDX2, and SATB2, in establishing a definitive diagnosis and differentiating CM from primary skin neoplasms. Early recognition of these lesions allows for more accurate staging, prognostication, and coordination of multidisciplinary care. Individualized treatment decisions should be based on the extent of the disease, the patient's performance status, and overall therapeutic goals. Further research and consensus development are warranted to establish standardized diagnostic and management strategies for CM across different cancer types.
